# The Value of Craft Beer Styles: Evidence from the Italian Market

**DOI:** 10.3390/foods12061328

**Published:** 2023-03-20

**Authors:** Francesco Bimbo, Emilio De Meo, Antonietta Baiano, Domenico Carlucci

**Affiliations:** 1 Department of Agricultural, Food, Natural Resources and Engineering Sciences (DAFNE), University of Foggia, Via Napoli, 25, 71122 Foggia, Italy; 2 Department of Soil, Plant and Food Science (Di.S.S.P.A.), University of Bari Aldo Moro, Via G. Amendola, 165/a, 70126 Bari, Italy

**Keywords:** craft beer, price variability, hedonic price model

## Abstract

This study aims to estimate the market value, or implicit prices, associated with the main craft beer attributes (e.g., beer style, organic, gluten-free, and package-related features) and support producers in detecting the more profitable marketing strategies. For this purpose, we conducted an empirical analysis employing sales data of craft beers from the Italian online market, and we estimated a hedonic price model via ordinary least squares. Results show that the type of package and cup only has moderate effects on price. Furthermore, a moderate premium price is found for gluten-free craft beers, while craft beers with organic labels and Italian origin do not benefit from the higher price. Instead, the beer style adopted strongly affected the product price: the highest premium prices were detected for Barleywine (+49.9%) and Italian Grape Ale (+39.6%) beer styles. Furthermore, relevant premium prices, higher than +25%, were estimated for other beer styles such as Sour, Fruit Beer, and Stout. Results suggest that artisanal breweries can effectively differentiate their product according to the beer style. To the best of our knowledge, the current study offers the first empirical evidence on how beer styles as well as other product characteristics affect the market price of craft beer by using secondary data.

## 1. Introduction

Beer is a fermented beverage with a long history dating back to the ancient civilizations of Egypt, Mesopotamia, China, and South America [1]. Over time, brewing techniques, based on the use of water, cereals, yeast, and hops, have been perfected and spread worldwide. Nowadays, beer is the most widely consumed alcoholic beverage in the world, in a much higher amount than wine, albeit with significant differences across countries [2].

During the 19th and 20th centuries, the global beer industry experienced the phenomenon of “industrialization” which saw the growth of only a few companies that benefited from the advantages related to technological progress and economies of scale [3]. In particular, at the end of the past century, a drastic process of market concentration was recorded, based on mergers and acquisitions, that led the global beer industry toward an oligopolistic structure with a few large multinational companies sharing most of the global market [3]. Within this context of the brewing industry, it was witnessed a progressive reduction of product differentiation because macro-brewers tend to choose product characteristics that appealed to as many consumers as possible [4]. As a consequence, beers offered by market-dominant multinationals became more and more homogeneous and standardized worldwide, with just one beer style (International Pale Lager) being dominant on a massive scale [5].

These market conditions facilitated the emergence of a new phenomenon that has been called the “craft beer revolution” and it consisted of the renaissance and rapid multiplication of small brewers offering more differentiated products capable to satisfy the increasing demand expressed by modern consumers for non-industrial, non-mass-produced food obtained from local raw materials and using local production methods [3,6]. These changes started in the 1980s on the west coast of the USA and rapidly spread to the rest of the country as well as all over the world [7]. Currently, small brewers with a broad differentiation capacity (also called “craft brewers”, “artisanal brewers”, “microbrewers”, “independent brewers”, “specialty brewers”, and “local brewers”) are playing a key role in transforming the global brewing industry [3]. Given the diversities among countries and their historically different traditions in beer brewing, a unique and generally accepted definition of craft beer does not exist. However, craft beer is usually identified from the producer’s perspective as beer brewed in any small, independently owned brewery that adheres to traditional brewing practices, so well distinguished from mass-produced beer offered by larger brewers [8].

In the last few years, many researchers investigated the phenomenon of craft beer from both the demand and supply sides. Two recent reviews highlighted that, in the last decade, hundreds of scientific articles addressing craft beer-related subjects have been published, most of them after 2017 [9,10]. Many of these studies have been conducted in different cultural contexts to analyze consumer preferences for craft beer characteristics and to identify the profile and motivations of craft beer drinkers [8,11,12,13,14,15,16,17,18,19,20,21]. These studies highlighted that two segments of beer drinkers may be distinguished regardless of the cultural context: “craft-style likers” and “mainstream-style likers”. The first consumer segment is smaller in the number of individuals than the second one, but it is rapidly growing. The segment of craft-style likers mostly includes males who are younger than their mainstream counterparts, and also have higher levels of income and education. From a psychological perspective, craft-style likers are characterized as having a strong self-identity that drives them to be more novelty-seekers and to have a desire for distinction by searching for products that are unique or, at least, well differentiated from mainstream beer. Craft-style likers are also highly involved with beer and they tend to regularly and consistently develop their knowledge by searching for and tasting new beers. In general, craft-style likers are willing to pay a higher price for beer and they enjoy a wide variety of beer styles and flavors.

In order to satisfy the needs expressed by this increasing group of consumers, microbrewers are growing exponentially in number, and they offer now a wide variety of craft beers that are highly differentiated in terms of both intrinsic and extrinsic characteristics [2]. Extrinsic characteristics of craft beer are mainly related to the packaging, brand, and labeling, in particular country-of-origin indication. As regard the intrinsic characteristics (i.e., color, alcohol content, flavor, smell, etc.), it is worth noting that craft beer can be obtained through a complex combination of different types and varieties of conventional beer ingredients (malted barley and wheat, unmalted cereals, hops, and yeast) with unconventional ones (fruits, spices, herbs, etc.), in different proportions and by using traditional and new brewing techniques, resulting in a wide range of beer styles [22]. Most importantly, many microbrewers are recovering traditional beer styles which were disappearing and now they reinterpret them in original and innovative ways [22]. However, although each craft beer may be considered an original and unique beverage, intrinsic characteristics of craft beer mainly vary according to the style which has been adopted and usually indicated on the label. Some organizations, such as The Brewers Association in the USA and Brewers of Europe, classified more than one hundred craft beer styles, and described each of them by color, clarity, perceived malt and hop aroma, bitterness, type of fermentation, alcohol content, etc. Other important intrinsic characteristics of craft beer concern the use of organic and gluten-free raw ingredients. 

The recent market trends have led to a significant increase in the variety of craft beers available on the market (hyperdifferentiation), and consumers highly demand products that precisely meet their needs and desires [23,24,25,26,27]. As a result of high-quality differentiation, the craft beer market is also characterized by high price variability, which reflects both different production costs and different consumer willingness to pay. For example, barrel-aged beer has a higher price firstly because barrel aging involves additional costs, and also because there are consumers who are willing to pay more for this type of beer.

This study starts with the observation that a wide variety of craft beers with different features and prices is now available on the market. Although it seems self-evident that the selling price of a product is consistent with the combination of the embedded attributes, in the market contexts where hyperdifferentiation and a wide range of prices exists, as in the case of craft beer, determining the relationship between the selling price of a product and its characteristics is not a simple matter. However, understanding whether and to what extent each quality attribute of craft beer affects the final price of the product, by generating a premium or a discount price, has important managerial implications. In fact, by knowing the economic benefit associated with a particular quality attribute (implicit price), producers could compare it with the relative costs incurred and so make more suitable product differentiation choices. In addition, producers would have the possibility to detect correct product pricing which plays a key role when highly differentiated products, such as craft beers, are introduced on the market. In fact, although consumers are willing to pay higher prices for craft beer, this does not exclude that consumers adopt a rational behavior and, among the various alternatives that meet their own expectations, they chose the one with a lower price. Therefore, the application of a too-high price may result in poor competitiveness of the product compared to other available alternatives, while a too-low price may be not sufficient to cover production costs and generate satisfactory profits. 

Thus, the specific purpose of this study is to estimate the market value, or implicit prices, associated with the most important attributes of craft beer and, in particular beer styles, each of which represents a specific combination of intrinsic attributes (color, alcohol content, flavor, smell, etc.) resulting from the use of specific raw materials and a specific brewing technique. 

To achieve this purpose an empirical analysis was carried out by considering the specific case of the Italian market and the e-commerce channel, which experienced extraordinary development during the period of restrictions due to the COVID-19 pandemic. Specifically, we employed a hedonic price model as an analytical tool for this investigation and we used data retrieved from a leading Italian online shop specialized in selling craft beer.

To the best of our knowledge, the current study offers the first empirical evidence on how beer styles as well as other product characteristics affect the market price of craft beer by using secondary data. The remainder of the article is organized as follows: Section 2 provides an overview of the Italian sector of craft beer by showing some general market data and a brief review of economic studies carried out in Italy; Section 3 describes the employed methodology; Section 4 discusses results; and Section 5 summarizes the main findings and highlights their marketing implications.

## 2. The Italian Craft Beer Market

### 2.1. Market Data

Italian beer production reached 1.76 billion L in 2021, surpassing both 2019’s (1.73 billion L) and 2020’s (1.58 billion L) production and it ranks in eighth place among EU producers [28]. Germany is the top European beer producer country with 7.5 billion L, followed by Poland (3.7 billion L), Spain (3.7 billion L), and the Netherlands (2.5 billion L). These four countries contribute 52% to the total EU beer production.

The Italian beer industry is highly concentrated. In 2021, the largest beer producers in Italy were Heineken Italia, with a market share of 33.7% (7031.3 hL), Birra Peroni, with a market share of 17.3% (3610.0 hL), Anheuser Busch Inbev Italia, with a market share of 9.5% (1978.0 hL), and Carlsberg Italia with a market share of 5.6% (1173.0 hL). Other popular beer brands in Italy are Birra Lucana, and Hausbrandt Trieste. Despite being dominated by a few large companies, the Italian beer industry is also characterized by a strong presence of craft beer producers. According to the Italian Brewers Association, there are approximately 650 craft breweries in the country, with a total production of 1.13 million hL in 2021. Craft beer account for 5% of total Italian beer production, employing over 5000 peoples as reported in Table 1 [28,29]. 

Data in Table 1 point out that Italy is quickly becoming an important player in the craft beer market. The craft beer industry has grown to include over 650 producers, with most of them located in the north of Italy. Italy’s craft beer scene has grown exponentially in the past few years, and the current market is estimated to be worth more than USD 100 million. More and more microbreweries are popping up all over the country, differentiating their beers from those of large producers, by offering a variety of styles and flavors. The most popular styles adopted by Italian craft beer producers include Amber Ale, Witbier, IPA, and Pale Ale. Many of these beers are brewed with local ingredients, such as chestnuts, honey, and spices. The Italian craft beer movement has seen a surge in popularity in recent years, with more people seeking out these unique, flavorful, and often local brews. Thus, although the Italian beer market is largely dominated by a few players producing a homogeneous product at a low price, there is a rising number of small and independent breweries which offer differentiated products capturing the growing interest of consumers for craft beers [28,29].

### 2.2. A Review of Italian Economic Studies on Craft Beer 

Studies investigating the Italian market of craft beer are still scant. Aquilani et al.’s work (2015) was the first study that investigated the emerging market of craft beer in Italy from a consumer perspective by conducting an exploratory survey involving “purely commercial beer drinkers” and “craft beer drinkers”; the authors observed a different profile in terms of some socio-demographic characteristics (i.e., age and professional status) and found that craft beer drinkers pay more attention to aroma, foam, carbonation, and the overall quality of beer; it was also observed that added flavors such as malted barley, chestnut, and honey increase the probability of perceiving craft beer to be of superior quality than commercial beer [11]. Donadini and Poretta (2017) conducted a conjoint rating experiment to estimate the importance attached by Italian consumers to different craft beer attributes and found that the greatest importance was placed on the type of container (glass bottle with a crown cap was the most preferred option) and on brewing technology (microfiltration was preferred because a clean craft beer was perceived better than a turbid one) [26]. Garavaglia and Mussini (2020) conducted a survey to investigate how Italian consumers interpret the meaning of “craft beer” and found that high value is assigned to craft products for their uniqueness, customization, originality, and personality; moreover, it turned out that craft beer is believed as produced by family-owned firms, in small-scale plants and it is non-pasteurized [25]. Carbone and Quici (2020) conducted an online survey associated with a choice experiment and found that, in general, Italian consumers are willing to pay more for craft beers rather than for industrial substitutes; furthermore, a specific segment of “craft beer enthusiast experts” was identified (30% of the analyzed sample) with better knowledge of craft beer and the highest willingness to pay [14]. Lerro et al. (2020) used the Best–Worst Scaling to detect consumers’ preferences towards several craft beer attributes and found that the most appealing craft beer attributes for Italian consumers are taste, fermentation process, color, and country of origin, while packaging material, brand, and price resulted to be the least interesting [20]. Rivaroli et al. (2022) investigated Millennials’ attitudes toward craft beer and found that the most important drivers of drinking craft beer are sensorial appeal, mood, and convenience mainly related to the possibility of purchasing craft beer online [21].

## 3. Materials and Methods

### 3.1. Data Collection and Description

The work employs data retrieved from the leading Italian online shop for craft beer “Cantine della birra” (https://www.cantinadellabirra.it, accessed on 4 July 2021). Since 2012, Cantina della birra is a specialized online store selling craft beer in the whole Italian market for consumers. Browsing Cantina della birra’s website in July 2021, we collected information on craft beers sold online and, for each one, its characteristics. In detail, we gathered information on the product price, whether the beer was produced according to organic practices, or whether it was gluten-free. Furthermore, we collected information on the package size and material, as well as whether the beer had a special cap (e.g., cork cap, tear-off cap) and whether produced by an Italian or foreign brewery. In addition, we collected information on the beer style used to produce the product and the brand. The product features collected from “Cantine della birra” website are listed in Table 2. The final database encompasses information on 1202 beers sold under 175 brands, produced in 17 countries, and using 28 beer styles.

The price of craft beers sampled ranged from 3.2 EUR/L to 23.93 EUR/L, with an average and median value of 12.50 EUR/L and 11.85 EUR/L, respectively. The majority (61.9%) of craft beers were packaged in glass bottles having a capacity equal to or lower than 0.33 L, the 64.89% of products in the sample. Instead, only a small share of beers in our sample, 6.66%, had special caps. 

Almost all the craft beers in our sample used raw materials obtained from conventional farming practices, 98.08% of products in our sample; the remaining share, 1.91% of products, was produced with organic ingredients and thus sold with organic labels. The data encompass gluten-free beers, which represent approximately 3% of product samples. Slightly less than 1 out of 2 beers in our data set was produced by an Italian brewery.

The most common beer styles were India Pale Ale, used to produce 28.45% of beer in our sample, then Sour (10.32%), Stout (7.32%), Strong Ale (6.99%), American Pale Ale (6.07%), and Abbey (5.32%). Instead, the less common beer styles were Dark Lager (0.83%), Italian Grape Ale (0.83%), Specialties (0.83%), Barleywine (0.75%), Herbal (0.67%), German Amber Lager (0.5%), Smoked (0.33%), Irish Red Ale (0.25%), Biere de Garde (0.17%), and California Common (0.08%).

### 3.2. Empirical Model and Statistical Analysis

In this work, we employed the standard hedonic price model proposed by Rosen (1974) [30]. According to the hedonic price framework, each consumer in the market selects a product having the optimal bundle of characteristics that maximizes his/her utility subject to a budget constraint. Likewise, each producer maximizes its profit by pricing the products sold given the attributes contained. In a market where each product represents a unique bundle of attributes, at the equilibrium, consumer willingness to pay for the product offered and producer willingness to accept the product sold will match, whose envelope will generate a hedonic price function [30,31]. 

The price of product *j* in market *m*, $P_{jm}$, can be described by the function:

(1) $$P_{jm}=f\left(X_{jm}\right)\;$$

where *X* is a vector of product characteristics and *f*(.) is an unspecified functional form. Equation (1) indicates that product price *j* in market *m* embeds the marginal monetary values of *j*’s attributes [31] and the marginal monetary value of *j* that can be obtained by partially differentiating (1) with respect to each attribute. In the current study, *X* encompasses beer features listed in Table 2 along with brand fixed effects. 

According to the previous literature (e.g., Carlucci et al., 2013; Szathvary and Trestini, 2013) [32,33], the parameters of Equation (1) are estimated using a single equation approach via ordinary least squares (OLS) with the dependent variable the logarithmic transformation of price and robust standard error for heteroskedasticity. Furthermore, the implicit price of each product feature was calculated using Kennedy et al.’s (1981) [34] adjustment proposed for dichotomic.

Estimates are reported in Table 3. In detail, the first column of Table 3 reports the variables included in the model; the second, the estimated parameters associated with each variable with standard errors in parentheses; and the third, and last column, reports the implicit prices of each product’s characteristics in percentage.

## 4. Results and Discussion

The estimated parameters of Equation (1) are reported in the first column of Table 3, along with their standard errors in parentheses, as well as the implicit prices of each product characteristic in percentage. 

The baseline product is a conventional Lager beer produced outside Italy, sold in glass bottles with a capacity of more than 0.33 L, having a crown cup, and sold at an average price of 12.50 EUR/L. The model shows an adjusted R^2^ equal to 0.8058 with a statistically significant value of the F-Statistic, indicating the joint significance of coefficient regressors. Ramsey’s RESET statistics for omitted variable bias had an F(3, 1018) value of 1.45 with a *p*-value of 0.212 [35], suggesting that the model does not suffer from misspecification. Furthermore, to discharge the potential multicollinearity issue in the model, we tested for multicollinearity via the variance inflation factor (VIF) test that provided a value of 4.88, below the threshold value of 10 above that existence of multicollinearity in the model is detected. The estimated coefficients for brand fixed effects are not reported in the manuscript for the sake of brevity and are available upon request.

**Figure 1:**
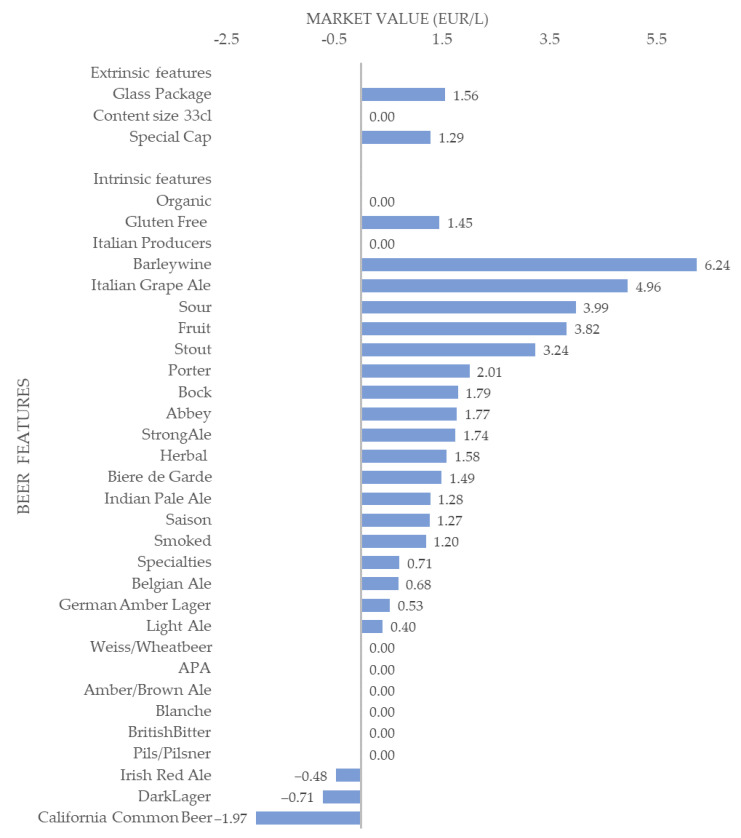
Market value of craft beer features.

First, the findings in Table 2, and graphically reported in Figure 1, show that the glass package material was associated with a premium price of +12.48%, or 1.56 EUR/L. This result points out the strong consumers’ preferences for craft beer packaged in glass bottles rather than in other packaging materials (tin can). Indeed, the glass packaging material is assessed as more suitable for craft beer as more hygienic, less polluting, and more taste-preserving [16]. However, the higher market price for glass-packaged beer products may reflect the higher cost of glass compared to that of other materials [36]. Package size is not associated with a premium price, while beer with a cork cap or tear-off cap benefits from a premium price of +10.32%, equal to 1.29 EUR/L, compared to the baseline product’s price having a crown cap. Such a result is partially in line with previous studies recording higher consumer interest in craft beers with cork caps compared with those having other kinds of caps. Cork caps contribute to providing a “premium image” to craft beer [16]. 

Organic beer is not associated with a premium price suggesting that consumers assess it as an “unnecessary” organic certification for beer. Thus, consumers are not willing to pay more than what they normally would spend to purchase conventional beer. Similar consumer bias against organic certification is recorded in the wine market [37]. However, our result contrasts a large bulk of recent evidence that consumers show a rising interest in a wide variety of organic products, especially when sold online [38,39,40,41]. Indeed, organic product sales from online platforms have grown during and after the COVID-19 pandemic, especially in several geographical contexts such as Poland and China, as well as among segments of the population such as Generation Y, born between the 1980s and 1990s. Generation Y, indeed, are “digital natives”, and appreciate all technological facilities to purchase food products (e.g., apps, price comparisons) as well as are interested in “bio”, “organic”, and “natural” products [42].

Instead, gluten-free beers record a premium price of +11.58%, corresponding to 1.45 EUR/L. The positive value of the gluten-free attribute is likely to be the result of the gluten-sensitive consumers’ high willingness to pay to enjoy beer without suffering from the side effects of gluten on health [43,44]. Surprisingly, Italian-produced beer did not receive a premium price compared to foreign alternatives. Indeed, such a finding contrasts recent evidence suggesting that consumers place a growing interest in local/domestic products by preferring the latter over foreign ones. Interest in local/domestic products has grown after the COVID-19 pandemic in order to support the recovery of local economies [45,46].

Second, estimated parameters reported in Table 3 show that the beer style affects the product’s price to a different extent, ranging from a premium of +49.95%, associated with Barleywine beers, to a price discount of −15.74% for California Common beers. However, it is worth saying that 18 out of 26 beer styles used to produce beers add value to the product, 6 out of 26 are not associated with a price premium, while only 3 out of 26 brewer styles are associated with a price discount. Such results suggest manufacturers may benefit from differentiating their products according to the style used to produce the beer. 

Results in Table 3 point out that Barleywine and Italian Grape Ale beer styles record the highest premium prices: the premium attached to such beer styles is +49.95%, or 6.24 EUR/L, and +39.65%, or 4.96 EUR/L, respectively (compared to the baseline option). The highest premium price associated with Barleywine beers is in line with the existing literature suggesting that such beer style is served for special occasions, holidays, or circumstances as “premium” beer [47]. Furthermore, Barleywine is a beer style preferred by connoisseur drinkers, in addition to a genuine enthusiasm for the style, it has become a status among craft beer fans. Barleywine beers indeed have peculiar features that originated from specific brewing techniques. In detail, Barleywine beers encompass a group of Strong Ales that rival the taste strength and complexity (a heavy malt backbone, accompanied by an intense mixture of flavors counterbalanced by a sturdy hop bitterness) and the alcohol content (from 6 to 12%). They were originally obtained through the so-called ‘parti-gyle’ method, applied to make the most of their ingredients. More specifically, brewers used massive amounts of malt and hops to create two or three separate styles of beer from a single mash. The first runs of wort were used for Stronger Ales, just the Barleywine beers, characterized by the highest level of alcohol and the suitability to be aged in wooden barrels. In the 20^th^ century, they began to be produced from a single brew, thus becoming a beer style and the name ‘Barleywine’ appeared on the labels [47]. Furthermore, the highest premium price associated with Barleywine beers may reflect the high taxation such beers are subject to and due to their high alcohol content, together with the higher production cost. Regarding Italian Grape Ale, its high premium price has already been recorded by Alfeo et al. (2019) [46] and related to its recognition as the first Italian-style beer [48]. Furthermore, the demand for high-alcohol content beers is likely to be highly inelastic; that is to say, when the price of such a product goes up its demand remains unchanged [49].

Then, Sour and Fruit beer styles, along with Stout style, record high premium prices ranging from +25.98% to +31.98% and corresponding to a monetary value ranging between 3 EUR/L and 4 EUR/L. Jaeger et al. (2019) [8] observed that fruity and full-flavored beers are preferred by craft beer drinkers, an ever-expanding market segment, who are known for their higher willingness to pay than the more traditional beer drinkers. Concerning Stout beer, it is a type of Strong Ale whose higher alcohol content and complexity respect a Lager beer already justify the positive premium price. Furthermore, Dark/Stout beers are among the most popular and preferred styles and their consumers are obviously willing to pay a premium to buy them [50,51].

Positive and significant impacts on craft beer price, between +9.58% and +16.09%, or from 1.2 EUR/L to 2.01 EUR/L, are provided by beer styles such as Smoked (+9.58%), Saison (+10.15%), Indian Pale Ale (+10.24%), Biere de Garde (+11.88%), Herbal (+12.64%), Strong Ale (+13.94%), Abbey (+14.19%), Bock (+14.35%), and Porter (+16.09%). The price premium associated with these beer styles is consistent with the evidence suggesting that consumers consider these styles novel for the Italian market and they are highly interested in trying them. Indeed, consumers described those beers as “unusual, intriguing, and complex” and whose consumption is appropriate for special occasions. Thus, consumers’ interest to try novel and intriguing beers as well as their consumption on special occasions justify the positive premium price we recorded for these beer styles [52].

Furthermore, the implicit prices associated with Specialties, Belgian Ale, German Amber Lager, and Light Ale beer stiles positively affect the craft beer price from +3.17% to +5.67%, adding a monetary value to the baseline product lower than 1 EUR/L. In detail, the marginal prices associated with specialty beers record a premium of +5.67%, Belgian Ale of +5.47%, German Amber Lager of +4.2%, and lastly, Light Ale of 3.17%. The parameters associated with these beers recorded a positive, albeit small, interest from Italian consumers. Thus, producers differentiating their products using such beer styles may record a marginal premium likely not enough to cover their production costs. 

Instead, brewery styles such as Weiss/Wheatbeer, American Pale Ale, Amber/Brown Ale, Blanche, British Bitter, and Pils/Pilsner do not affect beer price as the associated estimated coefficients are not significantly different than zero. These beers greatly differ from each other in physical, chemical, and sensory characteristics. Estimates for these beers, including Pils/Pilsner among the many, could be due related to the fact that such beers are perceived as appropriate in a broad range of contexts, and are also considered ordinary, simple, and boring [50,53]. Thus, consumers are unwilling to recognize a premium price for such beer styles.

Lastly, a discount price of −3.81% (−0.48 EUR/L) was found for Irish Red Ale brewery style, −5.72% (−0.71 EUR/L) for Dark Lager, and −15.74% (−1.97 EUR/L) for the California Common beer styles. A potential explanation for the price discount associated with these beers could be related to their color: red and dark for the first two styles, respectively, and ranging from medium amber to light copper color for the California Common [50]. Studies pointed out as beer color is a good predictor of consumer interest, though in a different manner depending on the cultural context. According to Donadini et al. (2016) [54], in the Italian market (the same market object of this study) dark and red-colored beers are less preferred compared to gold-colored ones. In detail, the authors found that Italians like less dark-colored beers than light ones, instead gold-colored beer positively affects consumer interest to buy and willingness to pay which is twice that recorded for beers having a red color.

## 5. Conclusions

This study starts with the observation that a wide variety of craft beers with different features and prices is now available on the Italian market. The aim of this study was to analyze the relationship between the market price and the main craft beer quality attributes. Data on the prices and characteristics of craft beer was retrieved from a leading online store offering a wide selection of Italian and foreign craft beers.

First, in the analyzed sample, which included over one thousand craft beers, we observed high price variability ranging from 3.2 EUR/L to 24.4 EUR/L, namely a maximum value eight times higher than the minimum. The applied hedonic price model provided a measure of the market value of the main extrinsic and intrinsic attributes of craft beers, namely the so-called implicit or shadow prices.

Second, the results of the analysis showed that extrinsic attributes of craft beer, namely the type of package and cup have only moderate effects on price. As expected, we detected a modest premium price for glass bottled craft beer (compared to canned products) and when a “special” cap is used (cork or tear-off cap instead of the common crown cap), while no difference in price was observed depending on the package size. 

Third, estimates for variables capturing the intrinsic beer characteristics pointed out a moderate premium price for gluten-free craft beers which, obviously, attracts the interest of those consumers who are gluten intolerant. Surprisingly, we did not detect any premium price for organic craft beers, unlike what is observed in other food markets where organic products usually receive a relevant premium price. Considering that the organic production method involves higher costs, this result can only be related to a little interest of consumers towards organic attributes associated with a product having a strong hedonistic value, such as craft beer. Furthermore, surprisingly, we do not have found any premium price for Italian craft beer with respect to foreign products contrary to what usually happens in other food markets where domestic products are sold at higher prices than imported ones because considered of better quality. 

Fourth, the analysis showed that the price of craft beer is strongly affected by the beer style adopted. It is worth remembering that 26 beer styles were represented in the analyzed sample and, compared to the mainstream Lager style, the highest premium prices were detected for Barleywine (+49.9%) and Italian Grape Ale (+39.6%). Relevant premium prices, higher than 25%, were also measured for other beer styles, specifically Sour, Fruit Beer and Stout. Fairly good premiums, higher than 10%, were observed for the following styles: Saison, Indian Pale Ale, Biere de Garde, Herbal Beer, Abbey, Bock, and Porter. Conversely, some beer styles gained only a rather modest price premium, lower than 10% (Specialties, Belgian Ale, German Amber Lager, Light Ale), and other styles (Weiss/Wheat Beer, American Pale Ale, Amber/Brown Ale, Blanche, British Bitter, Pils/Pilsner) did not record any significant difference in price compared to the baseline. Finally, some beer styles (i.e., Irish Red Ale, Dark Lager, California Common Beer) have even obtained a discount price.

The main managerial implication from our results is that microbreweries, at least those intending to sell on the Italian market, have to orient their production toward the adoption of some specific beer styles that appear to be more profitable than others, obviously by reinterpreting them in an original way. In particular, the results of this study suggest that more profitable styles are Barleywine, Italian Grape Ale, Sour, Fruit Beer, and Stout but it is possible also include Saison, Indian Pale Ale, Biere de Garde, Herbal Beer, Abbey, Bock, and Porter. Obviously, it must be carefully taken into account that real profitability depends on the price the product gains on the market but also on the occurred production costs which vary according to the adopted beer style. So, in order to make better strategic choices, producers should compare the benefit associated with a particular beer style (implicit price) with the relative costs incurred.

In spite of our results’ usefulness to provide guidance to craft beer producers in detecting more profitable marketing strategies, our analysis has some main limitations that may be overcome through future follow-up research.

Firstly, our findings refer specifically to the Italian market and therefore it would be desirable that they could be tested also in other countries. Secondly, we used data sampled from the main Italian online shop. Thus, future research could be aimed at obtaining estimates by expanding the number of online stores sampled, as well as by collecting data from physical beer shops and brew pubs. Thirdly, our estimates reflect the average contribution of craft beer features, including brewing styles, on price. However, the current analysis does not allow for non-linearities or combined effects of the attributes on prices. Thus, future research could use detailed household-level purchase data, flexible models, and estimation techniques (e.g., quantile regression) to tackle such limitations. Fourthly, a comparison of premium prices and relative costs associated with each attribute would offer a better picture of the actual mark-up achievable by craft beer producers that adopt product differentiation strategies. Lastly, our findings do not provide insights into the role played by consumers’ heterogeneity in the process of price formation, as obtained using aggregate market-level data. Therefore, a more in-depth analysis may be conducted to investigate consumer preferences and willing-to-pay-for craft beer attributes by using stated preference elicitation methods.

## Figures and Tables

**Table 1:** Italian beer industry data.

	Year											% Var. 2011–2021
	2011	2012	2013	2014	2015	2016	2017	2018	2019	2020	2021	
Breweries (n.)	350	421	509	599	688	757	868	874	853	769	814	132
Artisanal or microbrewers	336	407	491	505	540	718	693	692	684	624	657	95
Production (million hL)	13.4	13.3	13.3	13.5	14.3	14.5	15.6	16.4	17.3	15.8	17.6	31
Direct employment (n.)	4500	4700	4800	5000	5350	5350	5470	5500	5700	5200	5300	17
Import (million hL)	-	6.2	6.2	6.2	7.1	7.1	6.4	7.0	7.4	6.3	7.0	
Export (million hL)	-	2.0	2.0	2.1	2.5	2.6	2.8	3.0	3.5	3.3	3.8	
Domestic consumption (million hL)	17.7	17.5	17.5	17.7	18.9	19.0	19.8	20.4	21.2	18.7	20.8	18
Per-capita consumption (L)	28.5	28	28.5	29	31	31	33	34	35	31	35.2	24

Source: Assobirra “Report 2021”, Microbirrifici.org.

**Table 2:** Summary statistics and variables description (Obs. = 1202).

Variables	Variables Description	Mean ^a^
*Price*	Beer price EUR/L	12.502 (min: 3.2–max: 23.93; s.d. 3.30)
*Glass_Package*	1 = beer in glass bottle	0.6190
*Content_size_33cl*	1 = beer in package size equal or less of 0.33 L	0.6489
*Special_Cap*	1 = beer with special cap (e.g., cork, tear-off cap)	0.0666
*Organic*	1 = beer with organic certification	0.0191
*Gluten_Free*	1 = beer with gluten-free certification	0.0275
*Italian Producers*	1 = beer produced in Italy	0.4459
*American Pale Ale*	1 = American Pale Ale beer style	0.0607
*Abbey*	1 = Abbey beer style	0.0532
*Amber/Brown Ale*	1 = Amber/Brown Ale beer style	0.0100
*Barleywine*	1 = Barleywine beer style	0.0075
*Belgian Ale*	1 = Belgian Ale beer style	0.0216
*Biere de Garde*	1 = Biere de Garde beer style	0.0017
*Sour*	1 = Sour beer style	0.1032
*Fruit*	1 = Fruit beer style	0.0108
*Herbal*	1 = Herbal beer style	0.0067
*Blanche*	1 = Blanche beer style	0.0333
*Bock*	1 = Bock beer style	0.0166
*British Bitter*	1 = British bitter beer style	0.0158
*California Common*	1 = California Common beer style	0.0008
*Dark Lager*	1 = Dark Lager beer style	0.0083
*German Amber Lager*	1 = German Amber Lager beer style	0.0050
*India Pale Ale*	1 = India Pale Ale beer style	0.2845
*Irish Red Ale*	1 = Irish Red Ale beer style	0.0025
*Italian Grape Ale*	1 = Italian Grape Ale beer style	0.0083
*Lager*	1 = Lager beer style	0.0358
*Light Ale*	1 = Light Ale beer style	0.0399
*Pils/Pilsner*	1 = Pils/Pilsner beer style	0.0266
*Porter*	1 = Porter beer style	0.0250
*Saison*	1 = Saison beer style	0.0333
*Smoked*	1 = Smoked beer style	0.0033
*Specialties*	1 = Specialties beer style	0.0083
*Stout*	1 = Stout beer style	0.0732
*Strong Ale*	1 = Strong Ale beer style	0.0699
*Weiss/Wheatbeer*	1 = Weiss/Wheatbeer beer style	0.0341

^a^ For all binary variables, the mean represents the percentage of observations showing a value of 1 and the standard deviation is omitted.

**Table 3:** Estimated parameters and percentage of the premium price.

Variable	*β*	Percentage Premium Price ^a^
*Glass Package*	0.118 *** (0.0153)	+12.48
*Content Size less than 0.33 L*	0.010 (0.0283)	
*Special Cap*	0.098 ** (0.0488)	+10.32
*Organic*	0.044 (0.0430)	
*Gluten-Free*	0.110 *** (0.0321)	+11.58
*Italy*	−0.028 (0.0584)	
*American Pale Ale*	0.026 (0.0166)	
*Abbey*	0.132 *** (0.0107)	+14.19
*Amber/Brown Ale*	0.015 (0.0121)	
*Barleywine*	0.405 *** (0.0487)	+49.95
*Belgian Ale*	0.053 ** (0.0199)	+5.47
*Biere de Garde*	0.112 * (0.0623)	+11.88
*Sour*	0.277 *** (0.0468)	+31.89
*Fruit*	0.266 *** (0.0304)	+30.55
*Herbal*	0.119 *** (0.0248)	+12.64
*Blanche*	0.009 (0.0160)	
*Bock*	0.134 *** (0.0405)	+14.35
*British Bitter*	0.007 (0.0150)	
*California Common Beer*	−0.171 *** (0.0135)	−15.74
*Dark Lager*	−0.058 *** (0.0081)	−5.72
*German Amber Lager*	0.041 *** (0.0119)	+4.21
*India Pale Ale*	0.097 *** (0.0136)	+10.24
*Irish Red Ale*	−0.038 ** (0.0168)	−3.81
*Italian Grape Ale*	0.334 *** (0.0228)	+39.65
*Light Ale*	0.031 ** (0.0135)	+3.17
*Pils/Pilsner*	−0.032 (0.0200)	−3.23
*Porter*	0.149 *** (0.0167)	+16.09
*Saison*	0.096 *** (0.0266)	+10.15
*Smoked*	0.091 * (0.0475)	+9.58
*Specialties*	0.055 * (0.0309)	+5.67
*Stout*	0.230 *** (0.0375)	+25.93
*Strong Ale*	0.130 *** (0.0145)	+13.94
*Weiss/Wheatbeer*	0.035 (0.0547)	3.60
*Constant*	2.0568 ***	

^a^ Adjustment made according to Kennedy (1981). *, **, and *** are 10, 5, and 1 percent significance levels.

## Data Availability

The data presented in this study are available on request from the corresponding author. The data are not publicly available due to the type of authorization for their collection by store managers.
